# Strong Inhibitory Effect, Low Cytotoxicity and High Plasma Stability of Steroidal Inhibitors of *N*-Methyl-D-Aspartate Receptors With C-3 Amide Structural Motif

**DOI:** 10.3389/fphar.2018.01299

**Published:** 2018-11-12

**Authors:** Santosh Kumar Adla, Barbora Slavikova, Hana Chodounska, Vojtech Vyklicky, Marek Ladislav, Pavla Hubalkova, Barbora Krausova, Tereza Smejkalova, Michaela Nekardova, Marketa Smidkova, Lenka Monincova, Radko Soucek, Ladislav Vyklicky, Eva Kudova

**Affiliations:** ^1^Institute of Organic Chemistry and Biochemistry, Czech Academy of Sciences, Prague, Czechia; ^2^Institute of Physiology, Czech Academy of Sciences, Prague, Czechia; ^3^Faculty of Mathematics and Physics, Charles University in Prague, Prague, Czechia

**Keywords:** neurosteroid, amide, NMDA receptor, plasma stability, structure-activity relationship

## Abstract

Herein, we report the synthesis, structure-activity relationship study, and biological evaluation of neurosteroid inhibitors of *N*-methyl-D-aspartate receptors (NMDARs) receptors that employ an amide structural motif, relative to pregnanolone glutamate (PAG) – a compound with neuroprotective properties. All compounds were found to be more potent NMDAR inhibitors (IC_50_ values varying from 1.4 to 21.7 μM) than PAG (IC_50_ = 51.7 μM). Selected compound 6 was evaluated for its NMDAR subtype selectivity and its ability to inhibit AMPAR/GABAR responses. Compound 6 inhibits the NMDARs (8.3 receptors (8.3 ± 2.1 μM) more strongly than it does at the GABAR and AMPARs (17.0 receptors (17.0 ± 0.2 μM and 276.4 ± 178.7 μM, respectively). In addition, compound 6 (10 μM) decreases the frequency of action potentials recorded in cultured hippocampal neurons. Next, compounds 3, 5–7, 9, and 10 were not associated with mitotoxicity, hepatotoxicity nor ROS induction. Lastly, we were able to show that all compounds have improved rat and human plasma stability over PAG.

## Introduction

**FIGURE 1:**
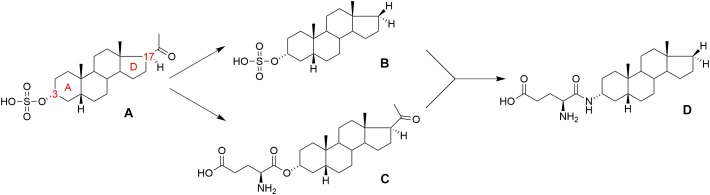
Structure of **(A)** pregnanolone sulfate (PAS), **(B)** androstane 3-sulfate, **(C)** pregnanolone 3-glutamate (PAG), and **(D)** PAG-like compound.

*N*-Methyl-D-aspartate receptors (NMDARs) are glutamate-gated ion channels, involved in excitatory synaptic transmission and synaptic plasticity (Citri and Malenka, 2008). These receptors are also associated with glutamate induced excitotoxicity under pathological conditions, which is a specific form of neuronal cell death caused by overactivation of NMDARs (Hynd et al., 2004; Dong et al., 2009). The activity of NMDARs can be influenced by allosteric modulators, including neurosteroids. One endogenous neurosteroid that inhibits responses of NMDARs (Petrovic et al., 2005) is 20-oxo-5β-pregnan-3α-yl sulfate (i.e., pregnanolone sulfate, PAS, Figure 1A).

In general, the major structural requirements for neurosteroid NMDAR-inhibitors have been established by several authors: (Irwin et al., 1994; Park-Chung et al., 1997; Weaver et al., 2000) detailing that the inhibitory effect is dependent upon both the 3α- and 5β-stereochemistry of the pregnane skeleton, in association with a negatively charged moiety at C-3, preferably a sulfate or hemiester group. As a part of our continuing interest in the structure-activity relationship (SAR) study of neurosteroid modulators of NMDARs, we have also recently reported on several new structural modifications that generate potent inhibitors of NMDARs: (i) the negatively charged C-3 substituent can be substituted by a positively charged moiety or zwitterion group (e.g., glutamic acid ester); (Borovska et al., 2012) (ii) the C-17 acetyl moiety of the pregnane skeleton is not essential for the inhibitory effect and can be substituted by non-polar substituents (methyl, ethyl, etc.), including full elimination of the C-17 moiety altogether, e.g., androstane 3-sulfate (Figure 1B); (Kudova et al., 2015) (iii) the steroidal D-ring is not essential for inhibitory effect and can be fully or partially degraded (Slavikova et al., 2016).

Pregnanolone glutamate (PAG, Figure 1C) (Borovska et al., 2012) was synthesized as the synthetic analog of PAS, and its neuroprotective effect was assessed in several biological models *in vivo*, wherein it was found that: (i) PAG does not induce psychotomimetic symptoms (such as hyperlocomotion and sensorimotor gating deficit), and it actually reduced excitotoxic damage of brain tissue and subsequent behavioral impairment in rats; (Rambousek et al., 2011) (ii) PAG significantly ameliorated neuronal damage in the dentate gyrus and subiculum, and improved behavioral performance in active allothetic place avoidance tasks (AAPA, also known as the carousel maze) after bilateral NMDA-induced lesions to the hippocampus; (Rambousek et al., 2011) (iii) PAG displayed anxiolytic-like and antidepressant-like properties in an elevated plus maze (EPM) and forced swimming models; (Holubova et al., 2014) (iv) PAG displayed a neuroprotective effect in a focal cerebral ischemia model that was induced by endothelin-1 in immature rats (Kleteckova et al., 2014).

**FIGURE 2:**
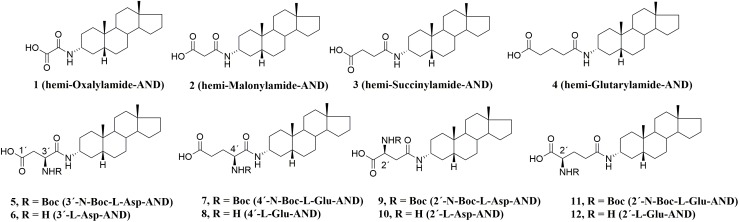
Pregnanolone 3-glutamate-like compounds 1–12.

From these literature reports, we concluded that the glutamic acid ester moiety could be a promising structural motif at the C-3 position for the development of drug-like molecules. However, we anticipated that the labile ester bond connecting the glutamate moiety of PAG to the steroid skeleton would be susceptible to plasmatic degradation by esterases. Thus, we thought to replace the ester linkage with the more robust amide bond (Figure 1D). This structural modification was designed to reduce the metabolic liability of new analogs and alter their solubility and permeability profiles. Moreover, the substitution of an ester bond by an amide has already been established as an effective isosteric approach that affords stable analogs while maintaining biological activity (Meanwell, 2011). Based upon our previous SAR on non-polar D-ring modifications, (Kudova et al., 2015) we also decided to incorporate this structural motif into our new library of NMDAR inhibitors. Hence, a series of amide-substituted PAG-like compounds was proposed (compounds 1–12, Figure 2).

From our previous studies, (Borovska et al., 2012; Kudova et al., 2014, 2015; Adla et al., 2017) we proposed that amide PAG-like compounds (1–12) offer a promising target for synthesis, further development and complex evaluation of physicochemical and biological properties including the screening on their stability in plasma. As such, compounds 1–12 were evaluated on HEK293 cells transfected with plasmids encoding GluN1-1a/GluN2B/GFP genes to elucidate the SAR of the amide, amino, and Boc-protected amino moiety in various positions. Considering the recommended guidelines for early stage development of new potent compounds, we have introduced mitotoxicity and hepatotoxicity screening on HepG2 cells as a primary tool to rank our compounds during lead optimization and to select the most promising candidate (Gerets et al., 2009; Van den Hof et al., 2013). From these results, we were able to identify lead compound 6, which we subsequently evaluated on recombinant GluN1/GluN2A-D receptors and native NMDARs α-amino-3-hydroxy-5-methyl-4-isoxazolepropionic acid (AMPA)/kainate receptors (AMPAR), and gamma-aminobutyric acid (GABA) receptors (GABARs) expressed in hippocampal neurons. Finally, we assessed the stability of PAG-like compounds vs. PAG in rat and human plasma to demonstrate their likely clinical advantage.

## Results and Discussion

### Chemistry

**FIGURE 3:**
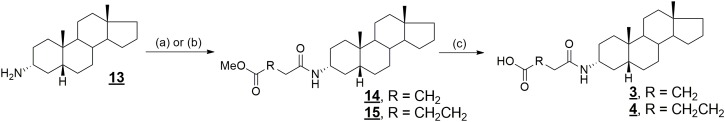
Synthesis of compounds 3 and 4. Reagents and conditions: **(a)** ClCO(CH_2_)_2_COOCH_3_, DIPEA, benzene, rt; **(b)** ClCO(CH_2_)_3_COOCH_3_, DIPEA, benzene, rt; **(c)** NaOH, THF, H_2_O, rt.

Compound 13 was prepared according to the literature: (Adla et al., 2017) in brief, commercially available 3β-hydroxy-5β-androstane was treated with phthalimide and triphenylphosphine, followed by deprotection of the amino group in hydrazine hydrate, to give 3α-amino derivative 13. Compounds 1 and 2 were prepared according to the literature (Adla et al., 2017) by treatment of compound 13 with the monoethyl ester of oxalic acid and methyl 3-chloro-3-oxopropionate, respectively, followed by basic hydrolysis. Analogously, compounds 3 and 4 were prepared by treatment of compound 13 with methyl 4-chloro-4-oxobutyrate and methyl 5-chloro-5-oxovalerate, respectively, affording compounds 14 in 79% and 15 in 83% yield. Finally, alkaline hydrolysis of compounds 14 and 15 afforded compounds 3 in 76% and 4 in 32% yield, respectively (Figure 3).

**FIGURE 4:**
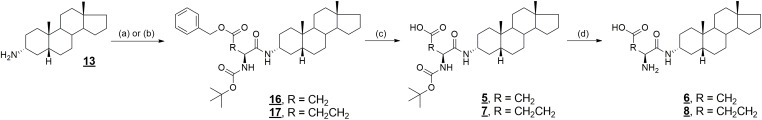
Synthesis of compounds 5–8 and 16, 17. Reagents and conditions: **(a)** Boc-Asp(OBzl)-OH, DIPEA, DMAP, DCC, benzene, rt; **(b)** Boc-Glu(OBzl)-OH, DIPEA, DMAP, DCC, benzene, rt; **(c)** Pd/C, H_2_, MeOH, rt; **(d)** TFA, CH_2_Cl_2_, rt.

The coupling of compound 13 with Boc-L-glutamic acid 5-benzyl ester and Boc-L-aspartic acid 4-benzyl ester, respectively, gave compounds 16 and 17 in 99% yield (Figure 4). Deprotection of the benzyl ether protecting group was achieved by hydrogenation catalyzed by palladium on carbon (compound 5, 65% yield and 7, 98% yield). Then, treatment of Boc-protected aspartate 5 and glutamate 7 with trifluoroacetic acid gave desired products 6 and 8 in 91% and 92% yield, respectively (Adla et al., 2017).

**FIGURE 5:**
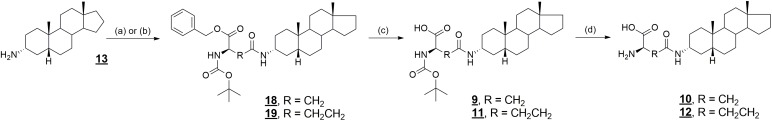
Synthesis of compounds 9–12 and 18, 19. Reagents and conditions: **(a)** Boc-Asp-OBzl, DIPEA, DMAP, DCC, benzene, rt; **(b)** Boc-Glu-OBzl, DIPEA, DMAP, DCC, rt; **(c)** Pd/C, H_2_, EtOH, rt; **(d)** TFA, CH_2_Cl_2_, rt.

Compounds 10 and 12 were prepared analogously to the synthesis of compounds 6 and 8, using Boc-L-glutamic acid 1-benzyl ester and Boc-L-aspartic acid 1-benzyl ester for coupling reaction with compound 13 (Figure 5). As such, compounds 18 and 19 were prepared in 93 and 92% yield, respectively. Then, deprotection of the benzyl ether protecting group was achieved by hydrogenation catalyzed by palladium on carbon (compound 9, 93% yield and 11, 96% yield). Finally, treatment of Boc-protected glutamates 9 and 11 with trifluoroacetic acid gave desired products 10 and 12 in 85% and 96% yield, respectively.

### Biological Activity

To investigate the activity of PAG and its amide analogs (1–12) on NMDARs, cDNA encoding for the rat GluN1-1a and GluN2B subunits were co-transfected into HEK293 cells. As the amphipathic character of compounds 1–12 is similar to our previously published D-modified steroids (Kudova et al., 2015), we have used an identical approach for the assessment of the obtained data and calculation of the IC_50_ values. In brief, the IC_50_ for the newly synthesized steroids was determined from a single dose of the steroid using the following formula:

$${\text{IC}}_{50}=[\mathrm{compound}]\times \sqrt[{h}]{\frac{1-I_{I}}{I_{I}}},$$

where *I*_I_ is the relative degree of inhibition, [*compound*] is the steroid concentration used, and *h* stands for the Hill coefficient (fixed at 1.2). The IC_50_ value was determined for a minimum of two steroid doses, differing twofold in the concentration range. If the difference in the IC_50_ values was <10%, then the mean steroid IC_50_ was calculated from the steroid concentration most proximal to that inducing 50% inhibition. If the difference in the IC_50_ values determined for two steroid doses was >10%, then the dose–response analysis was determined at lower steroid concentrations to reach the formal criterion. In accordance with previous results, IC_50_ calculations were made assuming 100% inhibition at saturating steroid concentration (Petrovic et al., 2005; Borovska et al., 2012). Table 1 summarizes the IC_50_ values determined for amide PAG-like compounds.

**Table 1:** Effects of compounds PAS, PAG (Figure 1), and 1–12 (Figure 2) on current responses of GluN1/GluN2B receptors in HEK293 cells to glutamate.

Receptor	IC_50_ ± *SD* (μM)	Conc. (μM)	*n*
PAS^a^	24.6 ± 5.3	100	5
PAG^b^	51.7 ± 8.6	200	7
1^c^	21.7 ± 4.8	10	7
2^c^	15.4 ± 3.5	10	6
3	11.2 ± 7.2	1	3
4	8.9 ± 1.0	1	3
5	5.4 ± 2.3	1	6
6^c^	4.8 ± 0.9	10	4
7	5.4 ± 2.4	1	6
8^c^	1.4 ± 0.2	3	6
9	4.5 ± 0.6	1	6
10	4.9 ± 1.7	3	6
11	3.4 ± 0.1	1	5
12	N/A^d^	N/A^d^	N/A^d^

*^a^See (Petrovic et al., 2005; Borovska et al., 2012); ^b^(Kudova et al., 2015); ^c^(Adla et al., 2017); ^d^Not soluble in 2.5 nor 5 mM DMSO.*

### The Ability of PAG-Like Compounds (1–12) to Modulate NMDAR Currents

Compounds 1–12 were evaluated for their inhibitory activity on NMDARs using HEK293 cells transfected with plasmids encoding genes for subunits of GluN1/GluN2B receptors. All compounds were established as more potent NMDAR inhibitors (IC_50_ values varying from 1.4 to 21.7 μM) than PAS (IC_50_ = 24.6 μM) and PAG (IC_50_ = 51.7 μM).

Compounds 1–4, bearing an alkylcarboxylic moiety of various chain lengths on the C-3 position, displayed relatively higher IC_50_ values (8.9–21.7 μM) as compared with the C-3 glutamate and aspartate derivatives 5–11 (1.4–5.4 μM). We also found that no significant difference in IC_50_ values was obtained for analogs with the transposed locations of the N-Boc and NH_2_ groups on the glutamate and aspartate substituent (3′ vs. 2′ for compounds 6 vs. 10 and 5 vs. 9). Lastly, we found that the introduction of a Boc-protecting group did not significantly alter the inhibitory effect of our compounds. Taken together, we can conclude that amide structural motif positively affects the ability of compounds 1–12 to modulate NMDAR currents.

### The Computational Estimate of Thermodynamic Properties of Compounds 1–12

The relevant physicochemical properties (Faassen et al., 2003) of the studied steroidal inhibitors were estimated by quantum mechanics computational methods and by a physicochemical properties predictor. The computational results are summarized in Table 2. We investigated the lipophilic qualities and solvation free energy (Δ*G*_solv_) of the inhibitors, as these properties are inherent characteristics of neuroactive compounds and influence their interactions with NMDAR (Kudova et al., 2015). The Δ*G*_solv_ values describe the behavior of single-molecules in water and in *n*-octanol, which acts as a proxy for the membrane environment. Lipophilicity of the compounds was estimated by the logP and logD coefficients.

**Table 2:** Summary of the computational values of the physicochemical properties of neuroactive steroids.

			Δ*G*_solv_ [kcal/mol] – transfer from					
			Vacuum to water		*n*-octanol to water			
Compound	IC_50_ [μmol/l]	Δ*G*_exp_ [kcal/mol]	Neutral	Charged	Neutral	Charged	logP	logD
1	21.7	−6.39	−13.25	−75.92	4.81	−3.96	3.96	0.67
2	15.4	−6.59	−17.76	−82.03	4.38	−5.01	3.73	0.96
3	11.2	−6.78	−17.03	−83.83	4.97	−4.51	4.29	0.98
4	8.9	−6.92	−18.42	−86.69	5.36	−4.64	4.56	1.52
5	5.4	−7.22	−21.21	−82.51	4.86	−3.59	4.37	1.64
6	4.8	−7.29	−17.90	−78.76	4.32	−4.50	3.70	0.86
7	5.4	−7.22	−23.89	−93.10	5.74	−4.78	3.33	2.01
8	1.4	−8.02	−20.22	−86.86	4.40	−5.42	3.85	1.14
9	4.5	−7.33	−21.79	−80.97	5.41	−3.92	4.76	1.38
10	4.9	−7.27	−21.78	−84.65	3.23	−5.96	2.82	0.85
11	3.4	−7.49	−25.50	−89.61	5.29	−4.52	4.80	1.71
12	N/A	N/A	−22.93	−85.84	3.56	−5.75	3.22	1.17

*The negative values of ΔG_solv_ signify the energy gained, and the positive values signify the energy required during the transfer from the first phase to the second phase. The experimental binding free energies (ΔG_exp_) were derived from the IC_50_ values via the equation ΔG_exp_ = RTln(IC_50_).*

The results in Table 2 show that the Δ*G*_solv_, logP and logD values of the investigated compounds are in the similar range to the earlier studied inhibitors (Kudova et al., 2015; Slavikova et al., 2016). Compounds 1–4 have more simple structures as compared to compounds 5–12 and evince a poorer inhibitory effect relative to the other studied compounds. For example, compound 1 with the simplest structure among the studied inhibitors (1–12) has the least inhibitory activity. The action of neuroactive compounds at the NMDAR is induced by a combination of many factors which determine the resulting inhibitory effect. Steroids interact with the entire non-polar inner surface of the NMDAR channel, mostly through attractive van der Waals interactions which compete with the repulsive effects (e.g., the partial desolvation at the site of the interaction, or the behavior of the charged and polar substituents of the steroid in the non-polar surroundings (Vyklicky et al., 2015). Previously, we demonstrated that the behavior of steroidal substances can be significantly influenced by conformational change inside the narrow region of the NMDAR channel, where the inhibitory effect occurs within proximity of the threonine ring through non-specific interactions (Vyklicky et al., 2015). Compounds 5, 7, 9, 11 (*N*-Boc protected) and 6, 8, 10, 12 (free NH_2_ group) have similar inhibitory effects, as well as similar Δ*G*_solv_, logP and logD values. It is evident that *N*-Boc addition does not improve the inhibitory effect. For instance, it can be caused by steric effect.

### *In vitro* Cytotoxicity of Compounds 1–12

In the present study, HepG2 cells were exposed to compounds 1–12 for 72 h. Then, the cell viability (XTT assay) and reactive oxygen species (ROS) induction were evaluated. The results of cytotoxic effect for PAG-like compounds (1–12) are summarized in Table 3. The hepatic effect of compounds 1–12 was compared with amiodarone (4.9 ± 0.2 μM) and nimesulide (2.2 ± 0.3 μM) – marketed drugs which cause hepatotoxicity.

**Table 3:** Mitotoxicity, hepatotoxicity and ROS induction in HepG2 cells of compounds PAS, PAG (Figure 1), and 1–12 (Figure 2).

Compound		Mitotoxicity (24 h, HepG2)^b^		Hepatotoxicity (72 h, HepG2)^b^	ROS induction^c^
		
	IC_50_ (μM) 25 mM glucose	IC_50_ (μM) 10 mM galactose	Glu/Gal index (fold change)	IC_50_ (μM) 5.5 mM glucose	EC_50_ (μM)
PAS	>200	> 200	n.d.	>200^a^	>200^a^
PAG	115 ± 13	58 ± 7	1.97	62 ± 9^a^	80 ± 11^a^
1	142 ± 12	72 ± 9	1.97	42 ± 7	52 ± 6
2	230 ± 29	174 ± 12	1.32	148 ± 14	165 ± 18
3	>200	>200	n.d.	>200	>200
4	48 ± 6	11 ± 3	4.27	4.0 ± 0.2	7.0 ± 2.0
5	>200	>200	n.d.	>200	>200
6	>200	>200	n.d.	>200^a^	>200^a^
7	>200	>200	n.d.	>200	>200
8	98 ± 11	94 ± 14	1.04	65 ± 5^a^	71 ± 9^a^
9	>200	>200	n.d.	>200	>200
10	>200	>200	1.22	>200	>200
11	193 ± 13	193 ± 17	1	91 ± 5	103 ± 8
12	N/A^d^	N/A^d^	N/A^d^	N/A^d^	N/A^d^
Amiodarone	63 ± 9	23 ± 5	2.70	4.9 ± 0.2	6.0 ± 1.0
Nimesulide	72 ± 8	18 ± 4	3.88	2.2 ± 0.3	3.5 ± 0.5

*^a^See (Adla et al., 2017). ^b^IC_50_ values indicate the concentration causing 50% decrease in cells viability using XTT cytotoxicity test (calculated using the non-linear regression method by GraphPad Prism). ^c^IC_50_ values indicate the concentration causing 50% increase of ROS detected using CM-H2DCFDA (calculated using the non-linear regression method by GraphPad Prism). ^d^Not soluble in 2.5 nor 5 mM DMSO.*

**FIGURE 6:**
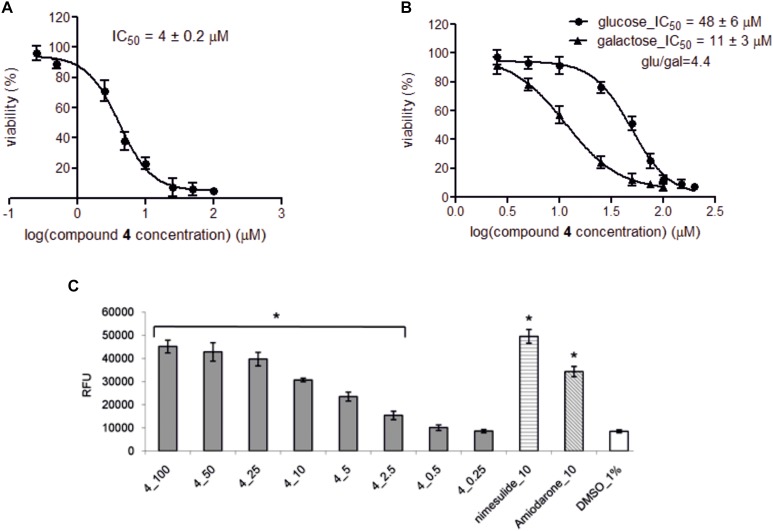
The effect of compound 4 on HepG2 viability and concentration-dependent effect of compound 4 and comparators on ROS level. **(A)** Hepatotoxicity provoked by compound 4 was assessed after 72 h incubation of HepG2 cells in low glucose medium (5 mM) in the presence of compound 4 (0.25–100 μM). **(B)** Mitochondrial toxicity was determined after 24 h incubation of HepG2 cells in either high glucose medium (25 mM) or galactose medium (10 mM) in the presence of compound 4 (2.5–150 μM). Cell viabilities were recorded in triplicates in three independent experiments and expressed as percentage of vehicle control ± SD and IC_50_ values were determined by GraphPad Prism software using following equation: *Y* = Bottom + (Top–Bottom)/(1 + 10ˆ((LogIC_50_-X)^∗^HillSlope)), where IC_50_ is the concentration of compound 4 that inhibits cell viability half way between Bottom and Top plateaus, X is compound 4 concentration and HillSlope describes the steepness of the family of curves. Glu/gal index higher than 3 indicates potential mitochondrial toxicity of compound. **(C)** Concentration-dependent effect on ROS level. HepG2 cells were treated with compound 4 (0.25–100 μM) for 72 h, and then the intracellular level of total ROS in relative fluorescence units (RFU) was detected. The data are presented as the mean ± SD for at least three independent experiments and each experiment was carried out in triplicate. Final concentration of DMSO in samples was 1%. Samples treated only with CM-H2DCFDA and 1% DMSO served as negative control, nimesulide and amiodarone (10 μM) serve as positive control. Single asterisks (^∗^) indicate a significant difference (*P* < 0.05) compared to 1% DMSO control (one-way ANOVA with Dunnett’s post-test).

Notably, compounds 3, 5–7, 9, and 10 displayed no adverse hepatic effect (>200 μM), whereas compounds PAG and 8 had a comparable hepatic effect (IC_50_ = 62 and 65 μM, respectively). Compound 4, bearing a five-carbon chain, had a strong hepatic effect (IC_50_ = 4.0 μM). Moreover, this compound showed Glu/Gal index higher than 3, which indicates its potential mitochondrial toxicity (Figures 6A,B). Interestingly, the adverse hepatic effect seen in compound 8 was abated by the inclusion of a Boc-protecting group on the glutamate moiety, as is reflected in matched compounds 7 (IC_50_ > 200 μM). Nevertheless, we strongly caution that a glutamate moiety at C-3 should be regarded as a red flag for cytotoxicity, warranting further research.

Contrary to the glutamate moiety, the aspartate moiety has been demonstrated as an “allowed” structural feature. Indeed, compounds 6 and 10, as well as their Boc-protected analogs 5 and 9, showed no adverse hepatic effect (>200 μM). Furthermore, compound 3, which has an analogous four-carbon moiety at C-3, also did not display any adverse hepatic effect (>200 μM). Therefore, we have established the aspartate moiety as a pharmacophore of the C-3 moiety to be further researched.

Decrease in cell viability was accompanied by concentration-dependent ROS induction (Figure 6C and Table 3). We hypothesize that the ROS mediated cytotoxicity can be associated with the type of side chain. Glutamate moiety, the source glutamate, has been reported to induce lipid peroxidation, decrease reduced glutathione and increase activities of catalase and superoxide dismutase in the liver of animals (Onyema et al., 2006). The hemioxalate moiety has been connected with lipid peroxidation (Sevam and Bijikurien, 1987). Shortening of chain from glutamate to aspartate, and extension of chain from oxalate to malonate did lead to loss of both ROS and cytotoxicity increase without decrease of inhibitory activity.

### The Inhibitory Effect of Compound 6 on GluN1/GluN2A-D Receptors

**FIGURE 7:**
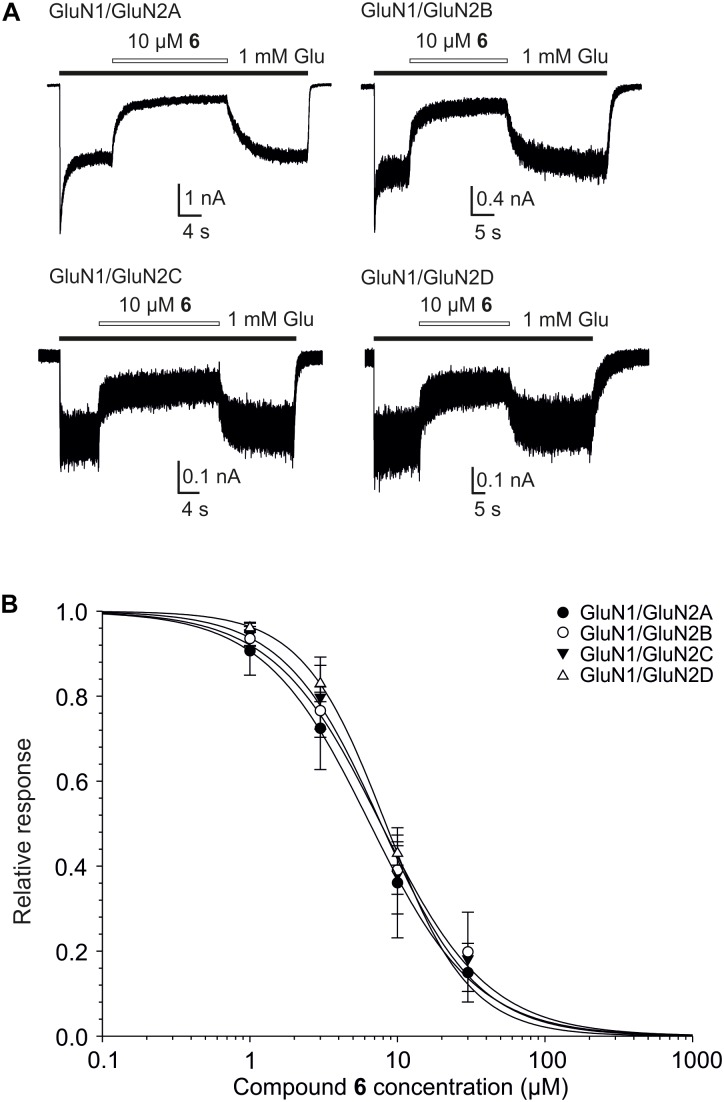
Concentration–response properties of compound 6 on GluN1/GluN2A-D receptors. **(A)** Examples of traces obtained from HEK293 cells transfected with cDNAs encoding GluN1/GluN2A, GluN1/GluN2B, GluN1/GluN2C, and GluN1/GluN2D receptors. Agonists (1 mM glutamate and 10 μM glycine) were applied alone and simultaneously with compound 6 (10 μM) to evaluate the inhibitory effect of compound 6 on NMDAR mediated currents (duration of glutamate and steroid application is indicated by filled and open bars, respectively). **(B)** Concentration–response curves for the compound 6 effect at GluN1/GluN2A-D receptors. Data points are averaged values of normalized responses from at least four HEK293 cells. Error bars represent SD. The relative agonist induced responses (*I*) recorded in the presence of compound 6 (1–30 μM) and determined in individual cells were fit to the following logistic equation: *I* = 1/(1 + ([*steroid*]/IC_50_)^h^), where IC_50_ is the concentration of steroid that produces a 50% inhibition of agonist-evoked current, [*steroid*] is the steroid concentration, and *h* is the apparent Hill coefficient.

Considering the effect of compounds 1–12 on current responses of GluN1/GluN2B receptors and their cytotoxicity profile, compound 6 (Figure 2) emerged as the lead structure and it was chosen for further biological evaluation. Comparison of the IC_50_ values of the steroid 6 at GluN1/GluN2A-D receptors shows no significant differences (one-way ANOVA; *P* > 0.05) (Figure 7 and Table 4) between NMDAR subtypes. This low subunit selectivity is strikingly different from previously published IC_50_ dependency of naturally occurring neurosteroid PAS on NMDAR subunit composition (Petrovic et al., 2005). PAS was found to inhibit GluN1/GluN2A-B (IC_50_ = 50.0 and 44.4 μM, respectively) receptors with lower potency than GluN1/GluN2C-D receptors (IC_50_ = 25.6 and 30.1 μM, respectively) (Petrovic et al., 2005). On the other hand, similar effect of compound 6 on NMDAR subunit dependency was found when compared to 17β-methyl analog of pregnanolone sulfate – 17β-methyl-5β-androstane 3α-yl-sulfate, which afforded comparable potency to all GluN1/GluN2A-D receptors (IC_50_ values varying from 0.4 to 0.7 μM). The reason for this phenomenon remains unknown.

**Table 4:** Inhibitory effect of compound 6 on GluN1/GluN2A-D receptors expressed in HEK293 cells and activated by 1 mM glutamate and 10 μM glycine.

Receptor	IC_50_ ±*SD* (μM)	*h* ± SD	*n*
GluN1/GluN2A	6.5 ± 0.7	1.3 ± 0.1	8
GluN1/GluN2B	7.4 ± 0.8	1.3 ± 0.1	5
GluN1/GluN2C	7.3 ± 0.8	1.6 ± 0.3	4
GluN1/GluN2D	8.4 ± 1.0	1.6 ± 0.3	4
one-way ANOVA^a^	*P* = 0.598	*P* = 0.066	

*^a^Statistical tests were performed for logIC_50_ and logHill values (one-way ANOVA); P ≤ 0.05 was used for the determination of significance.*

### The Effect of Compound 6 on Native NMDARs, AMPARs, and GABARs

**FIGURE 8:**
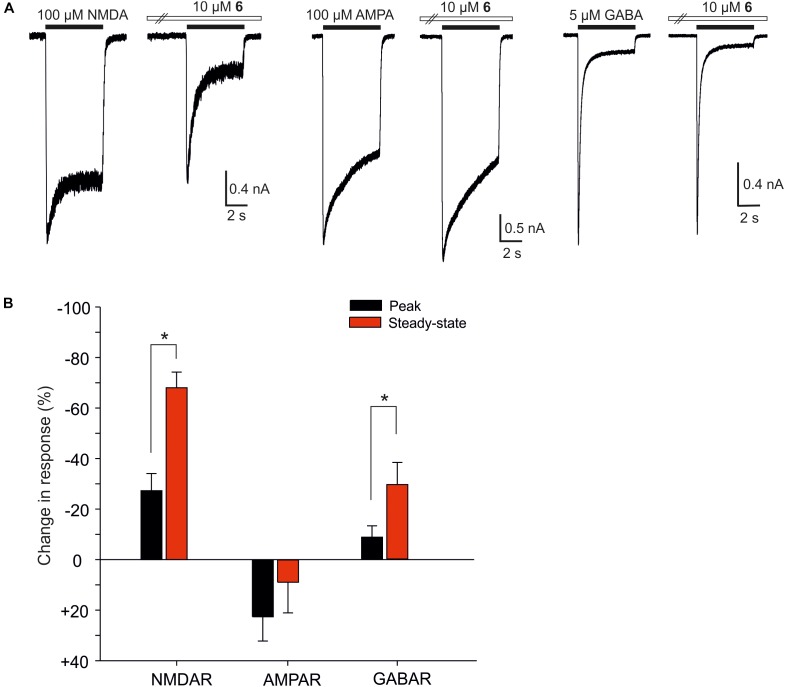
The effect of compound 6 on peak and steady-state responses of native NMDARs, AMPARs, and GABARs. **(A)** Examples of traces obtained from cultured hippocampal neurons. Compound 6 (10 μM) was pre-applied in the absence of agonists followed by co-application with NMDA (100 μM), AMPA (100 μM), and GABA (5 μM) (duration of agonist and steroid application is indicated by filled and open bars, respectively). **(B)** Bar graph represents the mean values of relative changes in the peak (black columns) and steady-state (red columns) responses of native NMDARs, AMPARs, and GABARs recorded in the continuous presence of 10 μM compound 6. Error bars represent SD from five independent measurements. Asterisks mark significance at the level of *P* < 0.05 (Paired *t*-test).

We have shown earlier that certain steroids preferentially inhibit tonically over phasically activated NMDARs with receptors with implications for synaptic transmission and excitotoxicity (Vyklicky et al., 2016). Figure 8 shows the analysis of the effect of compound 6 on the peak and steady-state response induced in cultured hippocampl neurons by fast application of NMDA, AMPA, and GABA. The data indicate that compound 6 present continuously was a more efficient inhibitor of tonically activated receptors at the steady–state of the response induced by co-application of the steroid with NMDA (100 μM) or GABA (5 μM) than of the peak response (Paired *t*-test; *P* < 0.001 and *P* = 0.009 for NMDARs and GABARs, respectively) (Figure 8B). The amplitude of responses induced by AMPA (100 μM) and recorded in the presence of cyclothiazide (10 μM) to block the receptor desensitization were only little affected by the steroid and no differences in the steroid effect at the peak and steady-state response were observed (Figure 8B).

**FIGURE 9:**
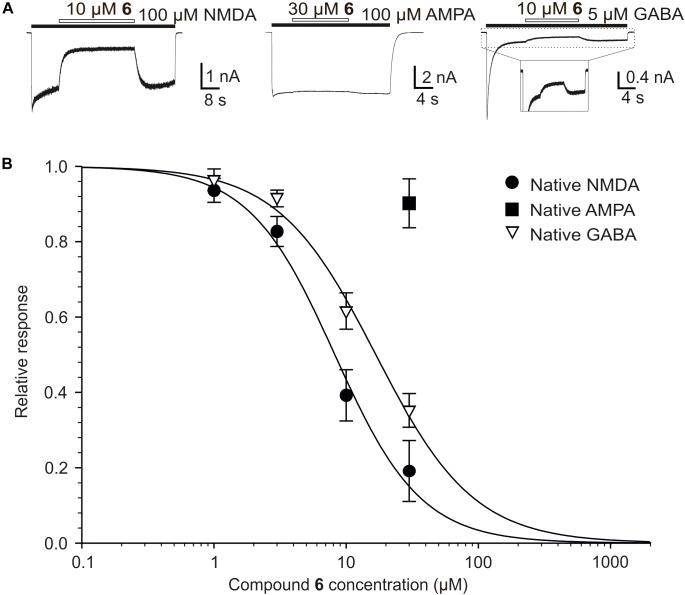
The effect of compound 6 on native NMDARs, AMPARs, and GABARs. **(A)** Example of traces obtained from cultured hippocampal neurons. Compound 6 (10 or 30 μM) was co-applied with NMDA (100 μM), AMPA (100 μM), and GABA (5 μM) (duration of agonist and steroid application is indicated by filled and open bars, respectively). **(B)** Concentration–response curves for the compound 6 effect at NMDARs and GABARs. Data points are averaged values of normalized responses from six cultured hippocampal neurons. Error bars represent SD. The relative agonist induced responses (*I*) recorded in the presence of compound 6 (1–30 μM) and determined in individual cells were fit to the following logistic equation: *I* = 1/(1 + ([*steroid*]/IC_50_)^h^), where IC_50_ is the concentration of steroid that produces a 50% inhibition of agonist-evoked current, [*steroid*] is the steroid concentration, and *h* is the apparent Hill coefficient. A low degree of inhibition induced by compound 6 in case of native AMPARs precluded detailed dose-response analysis.

We have also found differences in the potency of compound 6 to inhibit native NMDAR and GABAR (Figure 9). Concentration-response analysis of the inhibitory effect of compound 6 at native NMDAR responses showed IC_50_ = 8.3 ± 2.1 μM and *h* = 1.3 ± 0.2 (*n* = 6). In contrast, compound 6 inhibits the responses to 5 μM GABA with IC_50_ = 17.0 ± 0.2 μM and *h* = 1.2 ± 0.1 (*n* = 6). The differences in IC_50_ of compound 6 at NMDARs and GABARs was significantly different (*P* < 0.001; Student’s *t*-test). We have reported an opposite effect for other neurosteroid analogs where steroids and steroid-like compounds inhibited the GABARs with the same or higher affinity than NMDARs (Kudova et al., 2015; Slavikova et al., 2016). AMPAR responses were only little affected by compound 6 and estimates of IC_50_ from the steroid effect at a single dose (30 μM) and with a Hill coefficient fixed at 1.2 indicate value of 276.4 ± 178.7 μM (*n* = 6). Such a weak inhibitory steroid effect on AMPARs was not observed earlier for PAS and its synthetic analogs (Kudova et al., 2015; Vyklicky et al., 2015; Slavikova et al., 2016).

### The Effect of Compound 6 on the Frequency of Action Potentials

**FIGURE 10:**
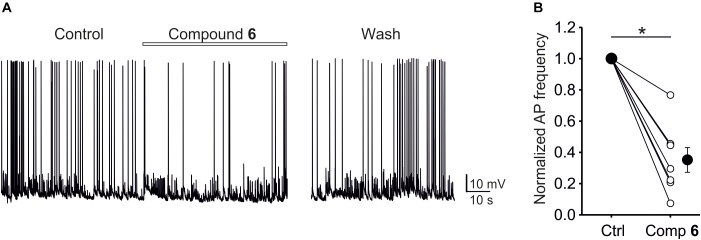
The effect of compound 6 on network activity in primary hippocampal cultures. **(A)** Example current-clamp trace showing control activity, activity in the presence of 10 μM compound 6, and following a period of wash. **(B)** Plot of normalized AP frequency before and during compound 6 application. Small circles show data from individual cells, large circles show mean data. Asterisks mark significance at the level of *P* < 0.001 (Paired *t*-test).

Given that steroids typically have multiple effects at the level of postsynaptic receptors, it is important to examine the net influence of a given compound on network activity. Compound 6, for example, potently inhibits NMDA receptors, but also shows inhibitory activity at GABA receptors. Therefore, we examined the effect of compound 6 (10 μM) on action potential firing frequency of neurons grown in primary hippocampal culture. Extracellular solution contained physiological concentrations of Ca^2+^ (2 mM), Mg^2+^ (1 mM) and glycine (10 μM). As shown in Figure 10, compound 6 consistently and robustly decreased the action potential frequency to 35 ± 8% of control values (Paired *t*-test, *P* < 0.001), and this effect was reversible. The observation that compound 6 has an overall inhibitory effect on network activity is consistent with its higher inhibitory potency at NMDA receptors compared to GABA receptors.

### Plasma Stability

As discussed above, PAG had not been expected to afford sufficient metabolic stability, as the glutamate moiety could undergo hydrolysis by carboxylesterase as described in the literature (Schötller and Krisch, 1974). Indeed, we have demonstrated that all compounds 1–11 have improved stability in rat and human plasma compared to PAG (Table 5) which confirmed our hypothesis of plasma-stable isosteric effect of amide structural modification.

**Table 5:** Stability of compounds PAG, 1–11 in rat and human plasma^a^.

Compound	Plasma stability (% remaining after 8 h)	
	Rat	Human
PAG	82	94
1	100	100
2	100	98
3	100	98
4	99	96
5	93	100
6	97	100
7	94	97
8	95	98
9	99	100
10	100	100
11	100	100

*^a^Data are reported as % of intact compound remaining following 8 h incubation.*

## Conclusion

In this study, we have examined a structural feature, the C-3 amide bond, which exhibits an inhibitory effect on NMDARs. Compounds 1–12 were evaluated for their ability to modulate NMDARs, wherein we demonstrated that the C-3 amide bond of these compounds is an allowed structural modification for maintaining inhibitory activity. Moreover, we have also shown that these new ligands are more potent than the endogenous ligand – PAS. In addition, we found that aspartate structural modification did not lead to an adverse hepatic effect while giving rise to improved plasma stability. Taken together, this new structural motif offers new prospects for the further modification and optimization of the pharmacological and pharmacokinetic properties of these neuroactive steroids.

## Experimental Section

### Chemistry

Melting points were determined on a micromelting point apparatus Hund/Wetzlar (Germany) and are uncorrected. Optical rotations were measured in chloroform using an Autopol IV (Rudolf Research Analytical, Flanders, NJ, United States). [α]_D_ values are given in deg (10^−1^deg cm^2^ g^−1^). IR spectra were recorded on a Bruker IFS 55 spectrometer (wavenumbers in cm^−1^). Proton and carbon NMR spectra were measured on a FT NMR spectrometer Bruker AVANCE-400 (400 MHz, 101 MHz) in CDCl_3_ with tetramethylsilane as the internal standard. Chemical shifts are given in ppm (δ scale). Coupling constants (J) and width of multiplets (W) are given in Hz. High resolution MS spectra were performed with a Q-Tof microspectrometer (Waters). Thin layer chromatography (TLC) was performed on silica gel (Merck, 60 μm). Preparative TLC (prep-TLC) was carried out on 200 mm × 200 mm plates coated with a 0.4 mm thick layer of the same material. For column chromatography neutral silica gel 60 μm (Merck) was used. Analytical samples were dried over phosphorus pentoxide at 50°C/100 Pa. The purity of the final compounds was assessed by a combination of NMR and on the basis of analysis LC-HR-MS, and the results were greater than 95%.

### Computational Section

#### Preparation of Structures

The studied compounds were manually built in PyMOL (version 1.5.0.4.) (De Lano and Lam, 2010) using the geometry of the molecule taken from the crystal structure (3CAV PDB code), (Faucher et al., 2008) and were relaxed by the RI-DFT/B-LYP/SVP method with the Turbomole program (version 6.1) (Ahlrichs et al., 1989). The empirical dispersion correction (D) (Jurecka et al., 2007) and COSMO continuum solvation model (Klamt and Schuurmann, 1993) were applied on the gradient optimization. The most stable local minima of the compounds were generated by the molecular dynamics simulation with the general AMBER empirical force field (the simulation was run 30 ns; the constant temperature was 400 K) (Wang et al., 2004). The AMBER 14 MD package was used (Case et al., 2014). The partial charges of the molecules were calculated using the RESP procedure (Bayly et al., 1993) at the HF/6-31G^∗^ level. The resulting geometries were minimized by the RI-DFT-D/B-LYP/SVP//COSMO method and their single-point energies (SP) were calculated at the RI-DFT-D3/B-LYP/TZVPP//COSMO level (Grimme et al., 2010). The chosen structures were re-optimized by the RI-DFT-D3/B-LYP/TZVPP//COSMO method and their SP were calculated at the same level of accuracy.

#### Computational Methods

The solvation free energy (Δ*G*_solv_) of the compounds was calculated in the SMD continuum solvation model (Marenich et al., 2009) (the transfer from vacuum to water and from *n*-octanol to water) at the HF/6-31G^∗^ level with the Gaussian program (version 09) (Frisch et al., 2009). In this method, the single-point energies were computed at the identical molecular geometry both for the transfer from vacuum to water and from *n*-octanol to water. The partition coefficient (P) is defined as the ratio of concentrations of a neutral solute in *n*-octanol and water, and it represents the solute lipophilicity. It is usually reported as common logarithm:

$$\log P=\log(c_{\mathrm{neutral},\mathrm{octanol}}/c_{\mathrm{neutral},\mathrm{water}})$$

The calculated logP was obtained via equation *logP* = Δ*G*_ow_/(−*RTln*(10)), where Δ*G*_ow_ is transfer free energy, *R* is molar gas constant and *T* is temperature (298.15 K) (Kolar et al., 2013; Bannan et al., 2016).

The Δ*G*_ow_ was calculated on the basis of the change in the molecular conformation related to the transfer between *n*-octanol and water. The compounds were optimized at the M06-2X/6-31G^∗^ level in SMD with the Gaussian, and Δ*G*_ow_ was expressed as the difference between the total energies in water and in *n*-octanol, because this energy includes the internal energy of the molecule (Kolar et al., 2013).

The distribution-coefficient (D), which is also presented as common logarithm, takes into account both neutral and ionized form of the solute in both phases and is used for estimation of lipophilicity of ionizable species (Kah and Brown, 2008).

$$\log D=\log\left(\frac{\left(c_{\text{ionized,octanol}}/c_{\mathrm{neutral,octanol}}\right)}{\left(c_{\text{ionized,water}}/c_{\mathrm{neutral,water}}\right)}\right)$$

The logD values were predicted at pH = 7.4, which is the physiological pH of blood serum, using the MarvinSketch program (ChemAxon, 2015).

### Biological Activity

Electrophysiological experiments were performed on HEK293 cells transfected with plasmids encoding rat GluN1-1a/GluN2A-D and GFP genes as described previously, (Petrovic et al., 2005; Cais et al., 2008; Korinek et al., 2010) or on primary hippocampal neurons prepared from P0–P1 rats, plated on collagen/poly-D-lysine-coated glass coverslips at a density of ∼50,000 cells/cm^2^, and grown in Neurobasal A medium with B27 Supplement (Gibco). Agonist-induced responses were voltage-clamped at a holding potential of −60 mV. Whole-cell voltage clamp recordings were made with a patch-clamp amplifier (Axopatch 200B; Axon Instruments Inc., Foster City, CA, United States) after a serial resistance (<10 MΩ) and capacitance compensation of 80–90%. For the application of test and control solutions, a microprocessor controlled multibarrel fast-perfusion system was used, with a time constant of solution exchange around cells of ∼10 ms. Agonist-induced responses were low-pass filtered at 2 kHz, digitally sampled at 5 kHz, and analyzed with pClamp software version 10.5 (Molecular Devices). Patch pipettes (3–5 MΩ) pulled from borosilicate glass were filled with Cs^+^-based intracellular solution (ICS) containing the following (in mM): 120 gluconic acid, 15 CsCl, 10 BAPTA, 10 HEPES, 3 MgCl_2_, 1 CaCl_2_, and 2 ATP-Mg salt (pH-adjusted to 7.2 with CsOH). The extracellular solution (ECS) contained the following (in mM): 160 NaCl, 2.5 KCl, 10 HEPES, 10 glucose, 0.7 CaCl_2_, 0.5 EDTA, and 0.01 glycine (pH-adjusted to 7.3 with NaOH). NMDAR responses were induced by 1 mM glutamate (in recombinant receptors) and 100 μM NMDA (native receptors). The ECS used for native receptors had the same composition as the ECS used for recombinant receptors. Native NMDAR and AMPAR recordings were performed on primary hippocampal mass-culture neurons at 8 days *in vitro*. For NMDAR recordings, ECS contained additionally 10 μM CNQX, 10 μM bicuculline and 0.5 μM TTX. AMPAR responses were induced by 100 μM AMPA and the ECS contained additionally 50 μM D-AP5, 10 μM bicuculline, 0.5 μM TTX, and 10 μM cyclothiazide. GABA receptor responses were induced in primary hippocampal mass-culture neurons at 12 days *in vitro* by 5 μM GABA and the ECS contained additionally 50 μM D-AP5, 10 μM CNQX, and 0.5 μM TTX. Action potentials were recorded in the current-clamp mode from primary hippocampal mass-culture neurons at 14–15 days *in vitro*. The ICS contained (in mM): 125 gluconic acid, 15 KCl, 5 EGTA, 10 HEPES, 0.5 CaCl_2_, 2 ATP-Mg salt, 0.3 GTP-Na salt, and 10 creatine phosphate (pH adjusted to 7.2 with KOH). The ECS contained (in mM): 160 NaCl, 2.5 KCl, 10 HEPES, 10 glucose, 2 CaCl_2_, 1 MgCl_2_, and 0.01 glycine (pH-adjusted to 7.3 with NaOH). Junction potential correction was applied *post hoc*. Steroid solutions were prepared fresh as a stock solution of either 5 or 20 mM in dimethyl sulfoxide (DMSO) before each experiment (1% DMSO final concentration). The same concentration of DMSO was added in all extracellular solutions. Experiments were performed at room temperature (21–25°C).

### HepG2 Cell Culture

HepG2 cells (ATCC^®^, United Kingdom) were grown in complete Minimal Essential Medium (EMEM) (5.5 mM glucose) medium, supplemented with 10% (v/v) fetal calf serum, 200 μg/mL of streptomycin, 200 U/mL of penicillin G and 4 mM glutamine. They were cultured under a humidified atmosphere containing 5% CO_2_ at 37°C.

### Cytotoxicity and Mitotoxicity Evaluation

The cytotoxicity of the tested compounds was assessed with use of XTT cell proliferation kit II (Roche Diagnostics GmbH, Mannheim, Germany) according to the manufacturer’s instructions. Briefly HepG2 cells were seeded in a 96-well plate at a density of 22,000 cells per well. After 24 h, the tested neurosteroids in a concentration range from 0.5 to 200 μM were added to the culture media and incubated for 72 h before the XTT dye was added. Mitochondrial toxicity was assessed in HepG2 cells using glucose and galactose conditioned media and XTT assay as a read out for cell viability. In brief, cells were seeded onto 96 well, clear bottom tissue culture plates at a density of 22,000 cells/well and left overnight to attach. Six hours prior to the neurosteroids treatment the media was replaced with 100 μL DMEM media containing either glucose (25 mM glucose; 1 mM pyruvate; 2 mM glutamine, 10% FBS) or galactose (10 mM galactose, 1 mM pyruvate, 6 mM glutamine, 10% FBS). After that, the tested neurosteroids in a concentration range from 0.5 to 200 μM were added in the fresh appropriate assay medium (glucose or galactose) and incubated for 24 h before the XTT dye was added. The XTT absorbance was recorded at 495 nm after 1 h incubation with the dye. Compounds were dissolved in DMSO to produce the stock solutions at the concentration of 10 mM, with exception of PAG, 1, 6 (5 mM), and 11 (2.5 mM). Results were expressed as percentages of change in viability compared to appropriate DMSO control. IC_50_ values were determined by GraphPad Prism version 5.00 for Windows (GraphPad Software, La Jolla, CA, United States). All experiments were done in triplicates in three independent experiments to check the reproducibility.

### Detection of Reactive Oxygen Species

The effect of neurosteroids on oxidative stress was determined using general oxidative stress indicator CM-H2DCFDA (Thermo Fisher Scientific, Waltham, MA, United States) according to the manufacturer’s instructions. Briefly HepG2 cells were seeded in a 96-well plate at a density of 13,500 cells per well. After 24 h, the tested neurosteroids were added to the culture media and incubated for 72 h. Subsequently, cells were washed with HBSS, treated with 100 μL of 1 μM CM-H2DCF-DA solution in HBSS and incubated for 1 h in the dark at 37°C. Finally, cells were washed three times with HBSS and the fluorescence was measured using 495 excitation and 527 emission in fluorescence microplate reader (Cytation 3, Winooski, VT, United States). IC50 index values were determined by GraphPad Prism version 5.00 for Windows (GraphPad Software, La Jolla, CA, United States).

### Plasma Stability

Compounds were prepared as 1.6 mg/mL stock solutions in methanol. Then, 37.8 μL of stock solution was added to 1400 μL of human plasma, maintained at 37°C. Aliquots (50 μL) withdrawn at 0, 1, 4, 8, and 24 h were analyzed by HPLC. An aliquot of plasma was extracted with methanol (450 μL) containing internal standard of deuterated pregnanolone glutamate (Rambousek et al., 2011) (5.3 μg/mL) and the solution was vortexed (20 s) and centrifuged at 13500 rpm for 10 min. The supernatant was transferred to an autosampler vial and 1 μL was injected onto LC-MS system. The samples were analyzed with an Agilent 1260 HPLC (Agilent Technologies) coupled to ESI-TOF Agilent 6530 (Agilent Technologies) with Agilent Jet Stream Technology. Samples were separated on a Waters ACQUITY UPLC CSH Phenyl-Hexyl (100 × 2.1, 130 Å, 1.7 μm) at a flow rate 0.3 ml/min. The concentration of mobile phase B (0.1% formic acid in acetonitrile) was gradually increased from 10 to 100% in mobile phase A (0.1% formic acid in water) over 7 min. The mass spectrometry instrument was operated in a negative ion mode with a voltage of +3.00 kV applied to the capillary. The temperature, the flow rate of the nitrogen drying gas, the pressure of the nitrogen nebulizing gas, and the flow rate of the sheath gas were set at 325°C, 10 l/min, 40 psi, 390°C, and 11 l/min, respectively. Results are represented as a percentage of a compound remaining in spiked human plasma.

### Experimental Data for Compounds 3–5, 7, and 9–19

#### General Procedure I –Coupling of Compound 13 With Protected Amino Acid

Protected amino acid (1.2 equiv) and DIPEA (1.6 equiv) were added at room temperature to a solution of compound 13 (1 equiv) in benzene (60 mL). Then, benzene (about 10 mL) was partially evaporated in vacuo and DMAP (7 mg) and dicyclohexylcarbodiimide in benzene (1M, 2.3 equiv) were added. The reaction mixture was stirred at room temperature and the progress was followed by TLC. Solvents were evaporated in vacuo and diethyl ether was added. The precipitate was filtered off and the filtrate was evaporated in vacuo.

#### General Procedure II – Hydrogenolysis of Benzyl Ether Protecting Group

Boc-Bn-conjugate with steroid was dissolved in ethanol (8 mL per 100 mg of steroid-conjugate). To this, Pd/C (10%, 10 mg per 100 mg of steroid-conjugate) was added. The reaction mixture was hydrogenated under slight pressure at room temperature for 10 h. Then, the reaction mixture was filtered through a short column of silica gel to remove the catalyst by washing with chloroform. Solvents were removed under vacuo.

#### General Procedure III – Boc-Group Deprotection

Trifluoroacetic acid (4 mL per 150 mg of Boc-protected compound) was added dropwise at room temperature to an ice-cooled solution of Boc-protected compound in dichloromethane (10 mL per 150 mg of Boc-protected compound). The reaction mixture was allowed to stir at room temperature until starting material was consumed (TLC monitoring). Then, the excess of dichloromethane and TFA was removed by flushing out with nitrogen. The resultant brownish oil was dissolved in pyridine/MeOH (4.5 mL/0.5 mL) and the solution was added dropwise to ice cold water (50 mL). The aqueous solution was kept in the fridge at 5°C for 24 h. The precipitate was filtered and dried in high vacuum to give the desired compound.

##### 2-(((3R,5R,8S,9S,10S,13S,14S)-10,13-dimethylhexadecahydro-1H-cyclopenta[a]phenanthren-3-yl)amino)-2-oxoacetic acid (1)

Compound 1 was prepared according to the literature (Adla et al., 2017).

##### 3-(((3R,5R,8S,9S,10S,13S,14S)-10,13-dimethylhexadecahydro-1H-cyclopenta[a]phenanthren-3-yl)amino)-3-oxopropanoic acid (2)

Compound 2 was prepared according to the literature (Adla et al., 2017).

##### 4-(((3R,5R,8S,9S,10S,13S,14S)-10,13-dimethylhexadecahydro-1H-cyclopenta[a]phenanthren-3-yl)amino)-4-oxobutanoic acid (3)

Compound 14 (37 mg, 0.095 mmol) and aqueous solution of NaOH (303 mg, 7.59 mmol, 10 mL) in THF (5 mL) were stirred for 18 h at room temperature. Then, the reaction mixture was poured into water (30 mL) and the pH was adjusted to 1 by adding aqueous aq. 5% HCl (30 mL). The product was extracted with ether (4 × 30 mL). The combined extracts were washed with brine (30 mL), dried over sodium sulfate and concentrated *in vacuo*. The residue was purified by preparative TLC (silica gel, 20–50% acetone in petroleum ether with 1% acetic acid) affording compound 3 (27 mg, 76%) as an amorphous solid: [α]_D_^20^ + 14.6 (*c* 0.43, CHCl_3_). ^1^H NMR (400 MHz, CDCl_3_): δ 0.70 (3H, s, H-18). 0.84-0.93 (1H, m), 0.96 (3H, s, H-19), 0.97–1.51 (14H, m), 1.52–1.79 (4H, m), 1.78–1.92 (2H, m), 2.50 (2H, t, *J* = 6.6 Hz, CH_2_CONH), 2.70 (2H, t, *J* = 6.6 Hz, HOOCCH_2_CH_2_), 3.74–3.83 (1H, m), 5.87 (1H, d, *J* = 8.1 Hz, NH) (Kleteckova et al., 2014). C NMR (101 MHz, CDCl_3_): δ 176.2 (CH2COOH), 171.6 (CH2CONH), 54.7, 49.9, 42.3, 40.9, 40.8, 40.5, 39.1, 36.1, 35.9, 34.7, 33.4, 29.7, 29.7, 27.7, 27.0, 26.8, 25.5, 23.6 (C-19), 20.8, 20.6, 17.5 (C-18). IR spectrum (CHCl_3_): 3515 (OH), 3432 (NH), 2932 (CH_2_), 2863 (CH_2_), 1714 (C = O), 1660 (amide), 1378 (CH_3_). MS ESI: *m/z* 398.3 (70%, M + Na), 376.3 (60%, M + H). HR-MS (ESI) *m/z*: For C_23_H_37_O_3_NNa [M + Na] calcd, 398.2666; found, 398.2666.

##### 5-(((3R,5R,8S,9S,10S,13S,14S)-10,13-dimethylhexadecahydro-1H-cyclopenta[a]phenanthren-3-yl)amino)-5-oxopentanoic acid (4)

Compound 4 was prepared in the same manner as described for compound 3. Starting from compound 15 (87 mg, 0.216 mmol), compound 4 was obtained (27 mg, 32%) by preparative TLC (silica gel, 20–50% acetone in petroleum ether with 1% acetic acid) as an amorphous solid: [α]_D_^20^ + 3.5 (*c* 0.172, CHCl_3_). ^1^H NMR (400 MHz, CDCl_3_): δ 0.73 (3H, s, H-18). 0.84–0.93 (1H, m), 0.99 (3H, s, H-19), 0.97–1.37 (10H, m), 1.42–1.50 (7H, m), 1.57–1.97 (8H, m), 2.20 (2H, t, *J* = 7.3 Hz, CH_2_CONH), 2.34 (2H, t, *J* = 7.2 Hz, HOOCCH_2_CH_2_), 2.86 (2H, br), 3.55–3.90 (1H, m), 6.91 (1H, br, NH), 10.70 (COOH) (Kleteckova et al., 2014). C NMR (101 MHz, CDCl_3_): δ δ173.4 (CH_2_COOH), 170.6 (CH_2_CONH), 54.6, 48.9, 42.5, 40.7, 40.7, 40.3, 38.9, 36.1, 36.1, 34.9, 34.6, 33.3, 32.6, 27.5, 26.9, 26.7, 25.9, 23.1 (C-19), 20.9, 20.6, 20.2, 16.9 (C-18). IR spectrum (CHCl_3_): 3608 (OH), 3455 (NH), 2930 (CH_2_), 2862 (CH_2_), 1721 (C = O), 1561 (amide), 1377 (CH_3_). MS (ESI): *m/z* 312.2 (70%, M + Na), 390.3 (45%, M + H). HR-MS (ESI) *m/z*: For C_24_H_40_O_3_N [M + H] calcd, 390.3003; found, 390.3004.

##### (S)-3-((tert-butoxycarbonyl)amino)-4-(((3R,5R,8S,9S,10S,13S,14S)-10,13-dimethylhexadecahydro-1H-cyclopenta[a]phenanthren-3-yl)amino)-4-oxobutanoic acid (5)

Compound 5 was prepared according to the literature (Adla et al., 2017).

##### (S)-3-amino-4-(((3R,5R,8S,9S,10S,13S,14S)-10,13-dimethylhexadecahydro-1H-cyclopenta[a]phenanthren-3-yl)amino)-4-oxobutanoic acid (6)

Compound 6 was prepared according to the literature (Adla et al., 2017).

##### (S)-4-((tert-butoxycarbonyl)amino)-5-(((3R,5R,8R,9S,10S,13S,14S)-10,13-dimethylhexadecahydro-1H-cyclopenta[a]phenanthren-3-yl)amino)-5-oxopentanoic acid (7)

Compound 7 was prepared according to the literature (Adla et al., 2017).

##### (S)-4-amino-5-(((3R,5R,8S,9S,10S,13S,14S)-10,13-dimethylhexadecahydro-1H-cyclopenta[a]phenanthren-3-yl)amino)-5-oxopentanoic acid (8)

Compound 8 was prepared according to the literature (Adla et al., 2017).

##### (S)-2-((tert-butoxycarbonyl)amino)-4-(((3R,5R,8S,9S,10S,13S,14S)-10,13-dimethylhexadecahydro-1H-cyclopenta[a]phenanthren-3-yl)amino)-4-oxobutanoic acid (9)

Compound 9 was prepared according to the General Procedure II – Hydrogenolysis of Benzyl Ether Protecting Group. Starting from compound 18 (810 mg, 1.40 mmol), compound 9 (656 mg, 96%) was obtained as white solid: mp 154–157 °C (Et_2_O), [α]_D_^20^ + 53.9 (*c* 0.28, CHCl_3_). ^1^H NMR (400 MHz, CDCl_3_): δ 0.68 (3H, s, H-18), 0.95 (3H, s, H-19), 1.44 (9H, s, O^t^Bu), 2.62–2.94 (2H, m, CH_2_CHCOOH), 3.69–3.86 (1H, m, H-3), 4.33 (1H, ddd, *J* = 10.0, 4.9, 2.3, BocNHCHCONH), 5.89 (1H, d, *J* = 4.8, BocNHCH_2_CONH), 6.36 (1H, bd, *J* = 7.8, CH_2_CONH) (Kleteckova et al., 2014). C NMR (101 MHz, CDCl_3_) δ 171.8 (COOH), 171.6 (CHCONH), 155.6 (CO, carbamate), 80.6 (CH_3_)_3_C-OC = O), 54.8, 50.8, 50.2, 42.4, 41.1, 41.1, 40.6, 39.2, 38.0, 36.2, 35.9, 34.8, 33.3, 28.4 (3x, (CH_3_)_3_COC(=O)NH), 27.4, 27.1, 26.9, 25.6, 23.6 (C-19), 20.9, 20.7, 17.6 (C-18). IR spectrum (CHCl_3_): 2977 (^t^Bu); 1744 (C = O); 1702, 1689 (carbamate, COOH); 1544, 1495 (amide); 1164 (O^t^Bu). MS ESI: *m/z* 489.3 (100%, M–1). HR-MS (ESI) *m/z*: For C_28_H_45_O_5_N_2_ [M–1] calcd, 489.33340; found, 489.33286.

##### (S)-2-amino-4-(((3R,5R,8S,9S,10S,13S,14S)-10,13-dimethylhexadecahydro-1H-cyclopenta[a]phenanthren-3-yl)amino)-4-oxobutanoic acid (10)

Compound 10 was prepared according to the General Procedure III – Boc-group Deprotection. Starting from compound 9 (150 mg, 0.31 mmol), compound 10 (102 mg, 85%) was obtained as white solid: mp 188–190°C (MeOH), [α]_D_^20^ + 20.4 (*c* 0.21, MeOH). ^1^H NMR (400 MHz, MeOD): δ 0.72 (3H, s, H-18), 0.98 (3H, s, H-19), 2.61 (1H, dd, *J* = 16.5, 9.3, HOOCCH(NH_2_)CH_2_CONH), 2.86 (1H, dd, *J* = 16.5, 3.6, HOOCCH(NH_2_)CH_2_CONH), 3.62-3.72 (1H, m, H-3), 3.80 (1H, dd, *J* = 9.3, 3.6, HOOCCHNH_2_), 7.89 (1H, s, HN). ^13^C NMR (101 MHz, MeOD) δ 173.0 (COOH), 171.3 (CHCONH), 55.9, 53.1, 50.8, 43.9, 42.1, 42.0, 41.5, 40.2, 37.5, 37.1, 36.2, 35.8, 34.1, 28.3, 28.2, 27.9, 26.5, 24.0, 21.9, 21.4, 17.8. IR spectrum (KBr): 3270 (NH); 1641 (amide); 1400 (COOH). MS ESI: *m/z* 389.2 (100%, M–1). HR-MS (ESI) *m/z*: For C_23_H_37_O_3_N_2_ [M-1] calcd, 389.28097; found, 389.28104.

##### (R)-2-((tert-butoxycarbonyl)amino)-5-(((3R,5R,8S,9S,10S,13S,14S)-10,13-dimethylhexadecahydro-1H-cyclopenta[a]phenanthren-3-yl)amino)-5-oxopentanoic acid (11)

Compound 11 was prepared according to the General Procedure II – Hydrogenolysis of Benzyl Ether Protecting Group. Starting from compound 19 (890 mg, 1.50 mmol), compound 11 (700 mg, 93%) was obtained as white solid: mp 125 – 128°C (Et_2_O), [α]_D_^20^ + 35.7 (*c* 0.29, CHCl_3_). ^1^H NMR (400 MHz, CDCl_3_): δ 0.68 (3H, s, H-18), 0.94 (3H, s, H-19), 1.45 (9H, s, O^t^Bu), 1.98–2.58 (4H, m, NHCOCH_2_CH_2_CHNH), 3.72–3.86 (1H, m, H-3), 4.19–4.29 (1H, m, HOOCCHNHBoc), 5.69 (1H, d, *J* = 6.5, BocNHCH_2_CH_2_CONH), 6.00–6.13 (1H, m, COHN). ^13^C NMR (101 MHz, CDCl_3_) δ 173.4 (COOH), 173.0 (CHCONH), 156.0 (CO, carbamate), 80.3 (CH_3_)_3_C-OC = O), 54.8, 53.2, 50.3, 42.4, 41.0, 41.0, 40.6, 39.2, 36.3, 35.9, 34.8, 33.3, 33.1, 29.5, 28.5 (3x, (CH_3_)_3_COC(=O)NH), 27.7, 27.1, 26.9, 25.6, 23.7 (C-19), 20.9, 20.7, 17.5 (C-18). IR spectrum (CHCl_3_): 2977 (^t^Bu); 1745, 1703 (C = O, COOH); 1658 (amide); 1163 (O^t^Bu). MS ESI: *m/z* 503.3 (100%, M–1). HR-MS (ESI) *m/z*: For C_29_H_47_O_5_N_2_ [M–1] calcd, 503.34905; found, 503.34845.

##### (R)-2-amino-5-(((3R,5R,8S,9S,10S,13S,14S)-10,13-dimethylhexadecahydro-1H-cyclopenta[a]phenanthren-3-yl)amino)-5-oxopentanoic acid (12)

Compound 12 was prepared according to the General Procedure III – Boc-group Deprotection. Starting from compound 11 (380 mg, 0.75 mmol), compound 12 (293 mg, 96%) was obtained as white solid: [α]_D_^20^ + 31.3 (*c* 0.24, MeOH). ^1^H NMR (400 MHz, CDCl_3_): δ 0.72 (3H, s, H-18), 0.97 (3H, s, H-19), 2.04–2.10 (1H, m, HOOCCH(NH_2_)CH_2_CH_2_CONH), 2.39 (1H, t, *J* = 7.2, HOOCCH(NH_2_)CH_2_CH_2_CONH), 3.57 (1H, t, *J* = 5.8, HOOCCH(NH_2_)CH_2_CH_2_CONH), 3.60–3.71 (1H, m, H-3), 8.52 (1H, s, HN). ^13^C NMR (101 MHz, MeOD): δ 174.0 (COOH), 173.7 (CHCONH), 55.9, 55.6, 50.8, 43.8, 42.1, 42.0, 41.5, 40.2, 37.5, 37.1, 35.8, 34.1, 33.3, 28.3, 28.1, 28.0, 27.9, 26.5, 24.0, 21.9, 21.4, 17.8. IR spectrum (KBr): 3275 (NH); 1637 (amide); 1400 (COO). MS ESI: *m/z* 403.3 (100%, M–1). HR-MS (ESI) *m/z*: For C_24_H_39_O_3_N_2_ [M–1] calcd, 403.29662; found, 403.29664.

##### (3R,5R,8S,9S,10S,13S,14S)-10,13-dimethylhexadecahydro-1H-cyclopenta[a]phenanthren-3-amine (13)

Compound 13 was prepared according to the literature (Adla et al., 2017).

##### Methyl 4-(((3R,5R,8S,9S,10S,13S,14S)-10,13-dimethylhexadecahydro-1H-cyclopenta[a]phenanthren-3-yl)amino)-4-oxobutanoate (14)

Methyl-4-chloro-4-oxobutyrate (41 μL, 0.318 mmol) in benzene (3 mL) was added dropwise at 0°C under inert atmosphere to a solution of compound 13 (66 mg, 0.212 mmol) and DIPEA (111 μL, 0.636 mmol) in benzene (5 mL). The reaction mixture was stirred at 0°C to room temperature for 18 h. Then, the reaction mixture was diluted with ethyl acetate (100 mL), washed successively with aqueous solution of sodium bicarbonate (2× 50 mL), brine (50 mL). The organic extracts were concentrated *in vacuo*. The residue was purified by column chromatography (silica gel, 20–50% ethyl acetate in petroleum ether) affording compound 14 (65 mg, 79%) as yellowish amorphous solid: ^1^H NMR (400 MHz, CDCl3): δ 0.70 (3H, s, H-18). 0.80 – 0.93 (2H, m), 0.95 (3H, s, H-19), 0.97–1.52 (14H, m), 1.51–1.77 (4H, m), 1.78–1.92 (2H, m), 2.45 (2H, t, *J* = 6.8 Hz, CH_2_CONH), 2.68 (2H, t, *J* = 6.8 Hz, CH_3_OCOCH_2_CH_2_), 3.70 (3H, s, OCH_3_), 3.74–3.83 (1H, m), 5.56 (1H, d, *J* = 8.0 Hz, NH). ^13^C NMR (101 MHz, CDCl_3_): δ 173.5 (CH_2_COOCH_3_), 170.4 (CH_2_CONH), 54.7, 51.8 (OCH_3_), 49.5, 42.4, 40.9, 40.8, 40.5, 39.1, 36.1, 35.9, 34.7, 33.6, 31.3, 29.6, 29.4, 27.9, 27.0, 26.8, 25.5, 23.58 (C-19), 20.8, 20.5, 17.5 (C-18). IR spectrum (CHCl_3_): 3435 (NH); 2932, 2863 (CH_2_), 1733 (C = O); 1663, 1516 (amide). MS ESI: *m/z* 412.3 (90%, M + Na), 390.3 (100%, M + H). HR-MS (ESI) *m/z*: For C_24_H_39_O_3_NNa [M + Na] calcd, 412.2822; found, 412.2819.

##### Methyl 5-(((3R,5R,8S,9S,10S,13S,14S)-10,13-dimethylhexadecahydro-1H-cyclopenta[a]phenanthren-3-yl)amino)-5-oxopentanoate (15)

Compound 15 was prepared in the same manner as described for compound 14 using methyl-5-chloro-5-oxobutyrate. Starting from compound 13 (100 mg, 0.32 mmol), compound 15 was obtained (107 mg, 83%) by column chromatography (silica gel, 20–50% acetone in petroleum ether) as a white amorphous solid: [α]_D_^20^ + 10.1 (*c* 0.187, CHCl_3_). ^1^H NMR (400 MHz, CDCl_3_): δ 0.70 (3H, s, H-18). 0.96 (3H, s, H-19), 0.96–1.52 (16H, m), 1.53–1.78 (7H, m), 1.78–1.93 (2H, m), 1.91–2.03 (2H, m), 2.21 (2H, dd, *J* = 7.9, 6.8 Hz), 2.40 (2H, t, *J* = 7.2 Hz, CH_3_OCOCH_2_CH_2_), 3.70 (3H, s, OCH_3_), 3.74–3.84 (1H, m), 5.42 (1H, d, *J* = 8.1 Hz, NH). ^13^C NMR (101 MHz, CDCl_3_) δ 173.7 (CH_2_COOCH_3_), 171.1 (CH_2_CONH), 54.7, 51.6 (OCH_3_), 49.3, 42.3, 40.9, 40.8, 40.5, 39.1, 36.1, 35.9, 35.7, 34.7, 33.7, 33.0, 27.9, 27.0, 26.8, 25.5, 23.5 (C-19), 21.0, 20.8, 20.5, 17.5 (C-18). IR spectrum (CHCl_3_): 3436 (NH); 2935, 2864 (CH_2_), 1731 (C = O); 1659, 1513 (amide). MS ESI: *m/z* 426.3 (40%, M + Na), 404.3 (100%, M + H). HR-MS (ESI) *m/z*: For C_25_H_41_O_3_NNa [M + Na] calcd, 426.2979; found, 426.2978.

##### (S)-benzyl 3-((tert-butoxycarbonyl)amino)-4-(((3R,5R,8S,9S,10S,13S,14S)-10,13-dimethylhexadecahydro-1H-cyclopenta[a]phenanthren-3-yl)amino)-4-oxobutanoate (16)

Compound 16 was prepared according to the literature (Adla et al., 2017).

##### (S)-benzyl 4-((tert-butoxycarbonyl)amino)-5-(((3R,5R,8R,9S,10S,13S,14S)-10,13-dimethylhexadecahydro-1H-cyclopenta[a]phenanthren-3-yl)amino)-5-oxopentanoate (17)

Compound 17 was prepared according to the literature (Adla et al., 2017).

##### (S)-benzyl 2-((tert-butoxycarbonyl)amino)-4-(((3R,5R,8S,9S,10S,13S,14S)-10,13-dimethylhexadecahydro-1H-cyclopenta[a]phenanthren-3-yl)amino)-4-oxobutanoate (18)

Compound 18 was prepared according to the General Procedure I – Coupling of Compound 13 with Protected Amino Acid. Starting from compound 13 (600 mg, 1.93 mmol), compound 18 (1.04 g, 93%) was obtained as a white amorphous solid by column chromatography (silica gel, 10% ethyl acetate in petroleum ether): mp 161 – 163°C (Et_2_O), [α]_D_^20^ + 23.8 (*c* 0.30, CHCl_3_). ^1^H NMR (400 MHz, CDCl_3_): δ 0.68 (3H, s, H-18), 0.93 (3H, s, H-19), 1.42 (9H, s, O^t^Bu), 2.61–2.91 (2H, m, NHCOCH_2_CHNHBoc), 3.72 (1H, tdt, *J* = 12.1, 8.5, 4.4, H-3), 4.53 (1H, dt, *J* = 8.9, 4.7, NHCOCH_2_CHNHBoc), 5.09–5.28 (2H, m, benzyl), 5.43 (bd, *J* = 7.4, BocHN), 5.79 (d, *J* = 8.5, COHN), 7.30–7.41 (5H, m, phenyl). ^13^C NMR (101 MHz, CDCl_3_) δ 171.4 (COOCH_2_Ph), 168.8 (CHCONH), 155.8 (CO, carbamate), 135.6, 128.6 (2xC), 128.4, 128.2 (2xC), 80.0, 67.4, 54.8, 50.7, 49.8, 42.5, 41.0, 41.0, 40.6, 39.2, 38.3, 36.3, 36.0, 34.8, 33.6, 28.4 (3x, (CH_3_)_3_COC(=O)NH), 27.9, 27.1, 26.9, 25.6, 23.7 (C-19), 20.9, 20.7, 17.6 (C-18). IR spectrum (CHCl_3_): 1742 (C = O); 1706 (carbamate); 1666, 1516, 1498 (amide); 1164 (O^t^Bu). MS ESI: *m/z* 603.4 (100%, M + Na), 581.4 (80%, M + 1). HR-MS (ESI) *m/z*: For C_35_H_52_O_5_N_2_Na [M + Na] calcd, 603.37684; found, 603.37685.

##### (R)-benzyl 2-((tert-butoxycarbonyl)amino)-5-(((3R,5R,8S,9S,10S,13S,14S)-10,13-dimethylhexadecahydro-1H-cyclopenta[a]phenanthren-3-yl)amino)-5-oxopentanoate (19)

Compound 19 was prepared according to the General Procedure I – Coupling of Compound 13 with Protected Amino Acid. Starting from compound 13 (600 mg, 1.93 mmol), compound 19 (1.05 g, 92%) was obtained as a white amorphous solid by column chromatography (silica gel, 10% ethyl acetate in petroleum ether): [α]_D_^20^ + 13.9 (*c* 0.25, CHCl_3_). ^1^H NMR (400 MHz, CDCl_3_): δ 0.68 (3H, s, H-18), 0.93 (3H, s, H-19), 1.43 (9H, s, O^t^Bu), 1.90–2.25 (4H, m, NHCOCH_2_CH_2_CHNHBoc), 3.74 (1H, dtd, *J* = 11.7, 7.6, 4.1, H-3), 4.25–4.35 (1H, m, NHCOCH_2_CH_2_CHNHBoc), 5.09–5.25 (2H, m, benzyl), 5.40 (1H, bd, *J* = 8.1, BocHN), 5.65 (1H, bd, *J* = 8.1, COHN), 7.30–7.41 (5H, m, phenyl). ^13^C NMR (101 MHz, CDCl_3_) δ 172.3 (COOH), 170.9 (CHCONH), 155.8 (CO, carbamate), 135.4, 128.7 (2xC), 128.6, 128.5 (2xC), 80.2, 67.3, 54.8, 49.6, 42.5, 42.4, 41.1, 41.0, 40.6, 39.2, 36.3, 36.0, 34.8, 33.6, 33.0, 28.7, 28.4 (3x, (CH_3_)_3_COC(=O)NH), 28.0, 27.1, 26.9, 25.6, 23.7 (C-19), 20.9, 20.7, 17.6 (C-18). IR spectrum (CHCl_3_): 2977 (^t^Bu); 1738 (C=O); 1709 (carbamate); 1661, 1506 (amide); 1163 (O^t^Bu). MS ESI: *m/z* 617.5 (100%, M + Na), 595.5 (60%, M + 1). HR-MS (ESI) *m/z*: For C_36_H_54_O_5_N_2_Na [M + Na] calcd, 617.39249; found, 617.39250.

## Author Contributions

Synthesis was done by SA, BS, and HCH. The inhibitory activity was evaluated by VV, ML, PH, BK, LV, and TS. The computational analysis was done by MN. Plasma stability was measured by LM and RS. Cytotoxicity, mitotoxicity, and ROS induction was measured by MS. EK wrote the manuscript.

## Conflict of Interest Statement

The authors declare that the research was conducted in the absence of any commercial or financial relationships that could be construed as a potential conflict of interest.
